# Human Plasmacytoid Dendritic Cells and Cutaneous Melanoma

**DOI:** 10.3390/cells9020417

**Published:** 2020-02-11

**Authors:** Matilde Monti, Francesca Consoli, Raffaella Vescovi, Mattia Bugatti, William Vermi

**Affiliations:** 1 Department of Molecular and Translational Medicine, University of Brescia, 25123 Brescia, Italy; m.monti002@unibs.it (M.M.); raffaella.vescovi@gmail.com (R.V.); bgtmtt@hotmail.it (M.B.); 2 Department of Medical and Surgical Specialties, Radiological Sciences and Public Health, Medical Oncology, University of Brescia at ASST-Spedali Civili, 25123 Brescia, Italy; francesca.consoli@icloud.com; 3 Department of Pathology and Immunology, Washington University School of Medicine, St. Louis, MO 63110, USA

**Keywords:** plasmacytoid dendritic cells, cutaneous melanoma, TLR

## Abstract

The prognosis of metastatic melanoma (MM) patients has remained poor for a long time. However, the recent introduction of effective target therapies (BRAF and MEK inhibitors for BRAFV600-mutated MM) and immunotherapies (anti-CTLA-4 and anti-PD-1) has significantly improved the survival of MM patients. Notably, all these responses are highly dependent on the fitness of the host immune system, including the innate compartment. Among immune cells involved in cancer immunity, properly activated plasmacytoid dendritic cells (pDCs) exert an important role, bridging the innate and adaptive immune responses and directly eliminating cancer cells. A distinctive feature of pDCs is the production of high amount of type I Interferon (I-IFN), through the Toll-like receptor (TLR) 7 and 9 signaling pathway activation. However, published data indicate that melanoma-associated escape mechanisms are in place to hijack pDC functions. We have recently reported that pDC recruitment is recurrent in the early phases of melanoma, but the entire pDC compartment collapses over melanoma progression. Here, we summarize recent advances on pDC biology and function within the context of melanoma immunity.

## 1. Introduction

The role of plasmacytoid dendritic cells (pDCs) in human pathology has been largely explored, mainly in autoimmune diseases [1]. Tumor-associated pDCs have also been identified almost two decades ago in solid tumors. However, their role during cell transformation and tumor progression is still controversial. Although, the function of type I interferon (I-IFN) is well-established in cancer immunoediting [2], the exact mission of pDCs in human cancer is still elusive. Here, we revise novel findings obtained from the recent literature as an extension to previously published reviews on the pDC biology [3,4,5,6,7], development [8], trafficking [9] and on their role in cancer [10,11]. More importantly, we review the recent findings on the role of pDCs during melanoma progression, with the proposal to provide the rationale for future treatment options.

## 2. Human Plasmacytoid Dendritic Cells: Biology and Functions

### 2.1. Development, Phenotype and Trafficking of Plasmacytoid Dendritic Cells

Plasmacytoid dendritic cells have been described, for the first time, by Karl Lennert [12] and subsequently characterized by Fabio Facchetti, as a distinct nodal immune cell populations [13,14,15]. In 1999 pDCs were found to correspond to the Natural Interferon Producing Cells, based on their ability to produce a large amount of interferon-α (IFN-α) in response to a variety of viral and synthetic stimuli [16,17].

**Figure 1:**
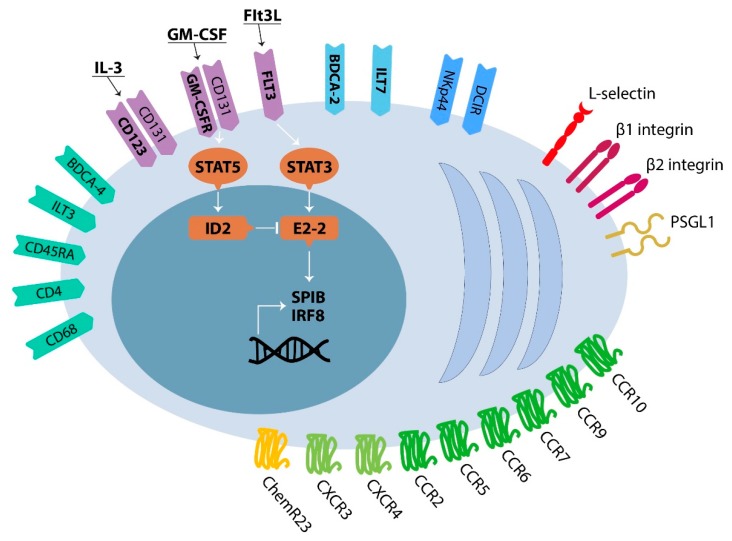
The phenotype of human pDCs. Graphical representation of the phenotype of a human pDC. Human pDCs express a broad range of surface antigens, adhesion molecules and chemotactic receptors. Among these, the surface receptors BDCA-2 and ILT7 are selectively express by human pDCs. Moreover, Flt3, GM-CSFR, and CD123 regulate the pDC development, homeostasis and survival via the ID2 and E2-2 transcription factors.

Circulating pDCs are a rare subset, corresponding to 0.2–0.8% of the total peripheral blood mononuclear cells (PBMCs). pDCs lack expression of the lineage markers specific for B cells, T cells, natural killer cells and myelo-monocytic cells. Human pDCs result negative for the myeloid dendritic cell (mDC) marker CD11c, as well. They can be identified based on their selective expression of surface antigens, such as the blood DC antigen 2 (BDCA-2/CD303; also known as C-type lectin CLEC4C) and the leukocyte immunoglobulin-like receptor subfamily A member 4 (LILRA4; also known as ILT7) [14]. Human pDCs also express BDCA-4 (CD304) [18], LILRB4 (also known as ILT3), CD45RA, CD4, CD68 and interleukin 3 receptor α-subunit (IL-3R/CD123) [19] (Figure 1). Accordingly, IL-3 mediates pDC survival in vitro [20]. In the peripheral blood, pDCs are defined as CD11c^−^ CD123^+^ CD303^+^ dendritic cells [21]. Human pDCs can be further classified into sub-populations with different phenotypes and functions [22,23,24,25,26]. Recently, three subsets of pDCs have been reported based on differential programmed death-ligand 1 (PD-L1) and CD80 expression in response to a single innate stimulus. Among these, i) PD-L1^+^CD80^−^ cells retain a plasmacytoid morphology and are specialized in I-IFN production; ii) PD-L1^-^CD80^+^ cells adopt a dendritic morphology and promote T cell activation with Th2 polarization; iii) PD-L1^+^CD80^+^ double positive pDCs have both innate and adaptive functions and an intermediate morphology [24]. Furthermore, different subsets of pDCs could be defined based on IFN-α or CXCL10 (also known as interferon-inducible protein 10; IP-10) expression [25,26]. Combining single-cell cytokine analysis with single-cell RNA-Seq profiling has demonstrated that the production of IFN-α by individually stimulated pDCs is controlled by stochastic gene regulation. Moreover, I-IFN amplification loop plays a major role in IFN-α response by pDCs [25]. Instead, the CXCL10^+^ and CXCL10^−^ subsets are defined by a distinct transcriptional program. This finding likely substantiates a diverse contribution of anti-viral responses and interferon-dependent inflammation [26].

The initial development of pDCs takes place in the bone marrow, from hematopoietic stem progenitor cells (HSPCs), and requires Fms-like tyrosine kinase 3 ligand (Flt3L) [27,28,29]. Terminally-differentiated pDCs are released from the bone marrow into the blood stream [30]. Human DC development is poorly understood, owing to the lack of an in vitro culture system that recapitulates in vivo DC hematopoiesis. Lee and colleagues have recently reported a culture system that supports the development of CD34^+^ HSPCs into the three major subsets of human DCs, combining Flt3L with mouse bone marrow stromal cells (MS5) [30]. Using this in vitro system, the sequential origin of human pDCs from increasingly restricted progenitors has been delineated. A human granulocyte-monocyte-DC progenitor (hGMDP) develops into a human monocyte-dendritic progenitor (hMDP) which in turn produces monocytes, and a human common DC progenitor (hCDP), the latter is restricted to conventional DCs (cDCs) and pDCs. Culture-derived pDCs showed a strong similarity with their in vivo peripheral blood counterpart both, in terms of gene expression profile and phenotype. hGMDPs, hMDPs and hCDPs are found in the bone marrow and cord blood of healthy individuals, but are undetectable in the peripheral blood and lymphoid organs.

Although many in vivo studies have focused on the pDC development within the myeloid lineage, pDCs can also be obtained from lymphoid progenitors. Recently, it has been demonstrated that human pDCs could arise from multipotent lymphoid progenitors (MLPs) and, even more, MLPs show better potential for pDC generation, compared to common myeloid progenitors (CMPs) [31]. Interestingly, the functions of CMP-derived and MLP-derived pDCs were found to be distinct in mice, for example in their ability to produce I-IFN and stimulate T cells [32]. In addition, after activation by influenza virus or CpG, human blood pDCs diversify into phenotypic and functional distinct subsets, which remain stable over time, demonstrating a further level of plasticity of this cell type [24]. For CDPs to pDCs commitment, up-regulation of the basic helix-loop-helix transcription factor E2-2 and the absence of the E protein antagonist inhibitor of DNA binding-2 (ID2) serve as key signals [33]. Second, pDCs have a low level of PU.1 and an extremely high concentration of interferon regulatory factor 8 (IRF8) and the ETS-family transcription factor SPIB [34,35,36]. Furthermore, a synergistic role of Flt3L and I-IFN is required to promote pDC development from mouse common lymphoid progenitors (CLPs) [37]. Once progenitors have committed to a pDC fate, the continuous expression of E2-2 maintains the pDC identity [35,38], and the high expression of SPIB controls the survival of pDCs and their progenitors through induction of anti-apoptotic gene BCL2-A1 [39]. Moreover, the signal transducers and activators of transcription STAT3 and STAT5 mediate the signals transduced through Flt3 and granulocyte-macrophage colony-stimulating factor receptor (GM-CSFR), regulating the balance between E2-2 and ID2 expression [40,41,42]. Flt3L promotes E2-2 expression through activation of STAT3, whereas GM-CSF induces the expression of ID2 via STAT5 activation (Figure 1). Recent technological advances have allowed the characterization of transcriptional profiles as well as in vivo lineage tracing at the single cell level, revealing that pDC commitment can be already imprinted in early lymphoid-primed multi-potential progenitors (LMPPs) and at the HSPC stage [43].

The molecular basis of tissue migration of pDCs have been largely studied and reviewed elsewhere [9]. Briefly, fully-differentiated pDCs reach the T cell areas of secondary lymphoid organs [13], mainly through the high endothelial venules (HEVs) [17], but they are nearly absent in the peripheral non-lymphoid tissues [14]. The recruitment of pDCs from the blood to peripheral tissues occurs under pathological conditions, such as inflammatory or neoplastic diseases [9]. The trafficking of pDCs is regulated by the action of adhesion molecules and chemokines (CKs). pDCs express L-selectin/CD62L, P-selectin glycoprotein ligand 1 (PSGL1), β1 and β2 integrins and chemokine receptors (CK-R) CXCR3, CXCR4, CCR2, CCR5, CCR6, CCR7, CCR9 and CCR10 [9] (Figure 1). Furthermore, pDCs express the chemerin receptor (ChemR23) and can migrate to lymph nodes, and at the sites of tissue damage in response to chemerin [44]. Upon pathogen encounter, pDCs can be directed to the lymph nodes by CCR7 and their secretion of inflammatory CKs may attract other pDCs, promoting cluster formation [45]. Additionally, CCR7 constitutively regulates the homing of pDCs on lymph nodes and splenic white pulp cooperating with CXCR4 [46,47].

### 2.2. The Type I and Type III Interferons Production by Plasmacytoid Dendritic Cells

**Figure 2:**
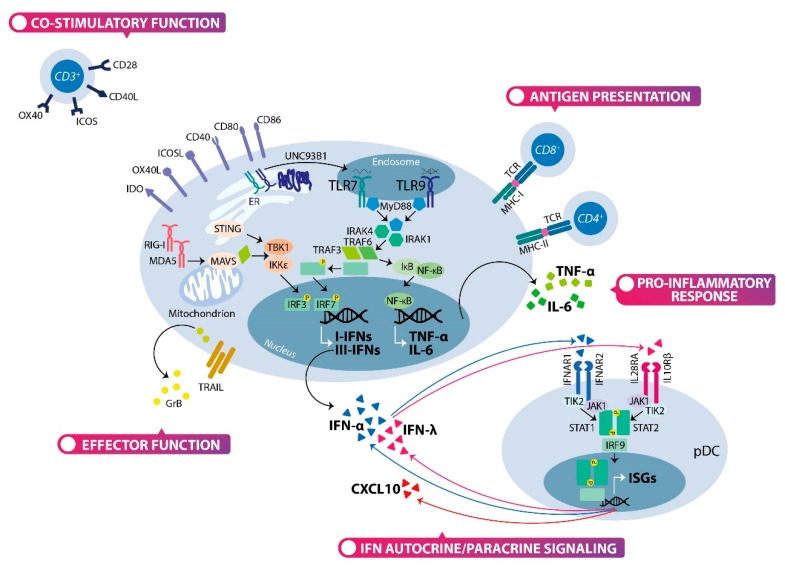
The multifaceted function of human pDCs. Graphical illustration of the human pDC functions. Human pDCs play a role in defense against pathogens, in autoimmunity and in cancer. pDCs perform these functions by modulating type I interferon and pro-inflammatory cytokines availability, by presenting antigens to T-cells, and by activating T-cells and exerting direct effector functions. Surface molecules and intracellular mechanisms involved are detailed.

pDCs represent a unique cell population within the immune system bridging the innate and adaptive immune responses [48], with documented roles in defense against pathogens, autoimmunity and cancer (Figure 2). Primarily, pDCs secrete large amounts of I-IFNs, in response to pathogens and self-nucleic acids [16,17]. Furthermore, pDCs are able to secrete other cytokines, including IL-6 and tumor necrosis factor-α (TNF-α), and pro-inflammatory CKs, such as IP-10. pDCs also express MHC-II and co-stimulatory molecules for antigens presentation.

The sensing of viruses or self-nucleic acids by pDCs is mainly mediated by endosomal Toll-like Receptor (TLR) 7 and 9 that rapidly induce the secretion of high levels of I-IFN, especially IFN-α [17,49]. It has been reported that IFNs account for 60% of transcripts in activated pDCs and transcriptional factors, critical for I-IFN (i.e., IFN regulatory factors; IRF) are preferentially expressed [50]. In addition, pDCs express the heterodimeric I-IFN receptor (IFNAR), formed of IFNAR1 and IFNAR2. Hence, they respond to IFN-α through the JAK/STAT pathway and the IRF8-mediated up-regulation of IFN-α autocrine/paracrine production [51,52], which promotes pDC survival via induction of anti-apoptotic genes [48]. On the other hand, the I-IFN feedback mechanism contributes to reduced pDC numbers during systemic viral infections, by activating the intrinsic apoptosis pathway [53], thereby, preventing an excessive inflammatory response and autoimmunity.

The TLR7 recognizes ssRNA viruses, endogenous RNA and synthetic oligoribonucleotides or imidazoquinoline compounds. Whereas, TLR9 detects DNA viruses containing unmethylated CpG-rich DNA sequences, endogenous DNA and synthetic CpG DNA. Viruses and endogenous nucleic acids reach the endosomal compartments in pDCs through autophagy or Fc receptors-mediated endocytosis as complexes with antibodies [54,55,56]. After TLR7/9 engagement, the IFN-α induction requires the activation of the myeloid differentiation primary response protein 88 (MyD88)-dependent pathway [57], as reviewed elsewhere in detail [6,58]. Despite the structural and functional similarities between TLR7 and TLR9, recent findings suggest that the activation of these receptors leads to different responses, depending on their intracellular localization. The chaperone UNC93B1 has a crucial role in controlling TLR7/9 trafficking from endoplasmic reticulum (ER) to endosomes in pDCs. In the endosome, TLR7/9 undergo proteolytic cleavage to attain the functional form. The activation of TLR7/9 in the early endosomes triggers the IRF7 signal cascade, mounting I-IFN response, whereas the engagement of TLR9 in the late endosomes triggers the NF-κB/MAPK signal cascade, promoting the production of pro-inflammatory cytokines and maturation [59,60]. Preventing the release of TLR9 from its trafficking chaperone within endosomes results in defective binding of its ligands and signal transduction, whereas TLR7 does not dissociate from UNC93B1 in the endosomes [61]. The phosphorylation of UNC93B1 mediates the recruitment of syntenin-1, a protein implicated in exosomes biogenesis, and promotes the TLR7 sorting to exosomes, limiting TLR7, but not TLR9 signaling [62]. This evidence, derived from UNC93B1 mutant, suggests that individual endosomal TLRs are distinctly regulated.

Human pDCs express a set of surface activating (FcγIIa and CD300a/c) and inhibitory (BDCA2, ILT7, NKp44 and DCIR) receptors that fine-tune the amplitude of I-IFN response and pDC activation state in response to TLR7/9 ligands. The counter regulation of TLR signaling is essential to prevent ongoing cytokine production implicated in autoimmune diseases. BDCA-2 cross-linking triggers the immunoreceptor tyrosine-based activation motif (ITAM) signaling pathway suppressing the ability of pDCs to produce I-IFN in response to TLR7/9 ligands [63,64,65]. The natural ligands of BDCA-2 have been identified as asialo-oligosaccharides with terminal galactose, normally present on cell surface glycoproteins, expressed by monocytes and, remarkably, by tumor cells [66]. Similarly, the receptor ILT7 associates with FcεRIγ and ILT7 cross-linking by anti-ILT7 mAb activates ITAM-mediated signaling cascade, which negatively regulates the TLR7/9 response in pDCs [67]. Bone marrow stromal cell antigen 2 (BST2), expressed by several human cancer cell lines, has been identified as the physiological ligand of ILT7 that inhibits the transcription and secretion of I-IFN and other pro-inflammatory cytokines by pDCs [68].

NKp44 is expressed on pDCs in tonsils and is induced on blood pDCs after in vitro culture with IL-3. NKp44 associates with DAP12 and NKp44 cross-linking inhibits I-IFN production by CpG DNA-activated pDCs [69]. NKp44 recognizes the proliferating cell nuclear antigen (PCNA) that is over-expressed in cancer cells [70]. DCIR is a C-type lectin receptor, containing intracellular immunoreceptor tyrosine-based inhibition motifs (ITIM) which binds glycans. The DCIR targeting inhibits TLR9-induced IFN-α production and results in antigen uptake and presentation by pDCs [71,72].

Although TLRs are the main innate receptors, involved in pDC activation, other cytoplasmic nucleic acid sensors are expressed by pDCs. RIG-I is expressed at low level in pDCs under steady-state conditions, but its expression can be greatly enhanced by endosomal TLR stimulation in I-IFN-independent manner [73]. RIG-I and the melanoma differentiation associated gene-5 (MDA-5) cellular helicases have been implicated as the dominant cytoplasmic receptors for dsRNA [74]. pDCs also express the NOD-like Receptors (NLRs), namely NLRC5 and NLRX1, in inducible, or constitutive manner, respectively, which negatively regulates the RIG-I-like receptors (RLRs)-induced I-IFN production in pDCs [75]. In addition, cyclic GMP-AMP (cGAMP) synthase (cGAS) - stimulator of IFN genes (STING) signaling pathway is expressed in human pDCs [76]. STING is an ER-associated protein required for the induction of I-IFN response, independent of TLR9, by sensing cytosolic DNA pathogens or cyclic dinucleotides [77]. Cytosolic DNA activates cGAS to form cGAMP, a cyclic dinucleotide that binds and activates STING. Subsequently, STING activates NF-κB and IRF3 through the kinases IKK, and TBK1, respectively. The transcription factors phospho-IRF3 and NF-κB translocate to the nucleus and induce the production of I-IFN with other cytokines [78]. However, it should be noted that MyD88-dependent I-IFN signaling pathway produces 100-fold higher cytokine amounts than IRF3-dependent I-IFN signaling pathway. Moreover, cGAS-STING- and MDA5-MAVS-mediated signaling induce the expression of SOCS1 that is a negative regulator of MyD88-mediated I-IFN signaling in pDCs [79].

Human type III IFNs include four subtypes: IFN-λ1 (IL-29), IFN-λ2 (IL-28A), IFN-λ3 (IL-28B) and the more recently discovered IFN-λ4 [80,81,82]. III-IFNs signal through the heterodimeric receptor IFNLR, formed of IL28Rα and IL10Rβ subunits. Even if the I-IFNs and III-IFNs bind to distinct receptors, they activate similar signaling pathways and transcriptional responses. Although, many similar genes are activated by both I- and III-IFNs, they have some distinct biological features. III-IFNs provide a first-line antiviral defense at anatomic barrier surfaces and is essential for innate mucosal immunity [83]. III-IFN response is less inflammatory and confers less collateral damage than the more potent I-IFN response. Relevantly, III-IFNs exert direct effects on cancer cells by inhibiting cell proliferation and promoting apoptosis. In addition, III-IFNs have indirect effects against multiple tumors by inducing T cells and NK cells response [84,85,86]. pDCs produce IFN-λ1/3 in response to either stimulation by viruses or synthetic TLR9 agonists, independently of IFN-α [87,88]. However, the pDC subset expressing IFN-λ corresponds to the cell subset with the highest level of IFN-α expression [88].

The expression of functional receptor, IFNLR, is restricted to epithelial cells and leukocyte subsets, including pDCs [83]. IFN-λ triggers STAT phosphorylation and interferon-stimulated genes (ISG) transcription. IFN-λ functions as autocrine/paracrine signal to sustain antiviral response by increasing IFN-α and IFN-λ production in pDCs [88]. Moreover, IFN-λ modulates the expression of co-stimulatory molecules (e.g., CD80, CD86), the maturation marker CD83 and other phenotypic markers (e.g., HLA-DR, CD123 CD303); it induces the CKs synthesis and prolongs pDC survival [89,90]. The role of IFN-λ in the regulation of pDC phenotype, survival and functions has been extensively review elsewhere [91].

### 2.3. The Multifaceted Function of Plasmacytoid Dendritic Cells: Not Only Interferon Producing Cells

In addition to their central role in interferon production, pDCs may exert direct effector functions. In particular, the activation of pDCs via TLR7/9 stimulation induces the expression of TNF-related apoptosis-inducing ligand (TRAIL) [22,92], which mediates the cell death of TRAIL-sensitive infected cells and tumor cells, expressing either TRAIL-R1 or TRAIL-R2 [93]. Autocrine IFN-α/β signaling also regulates TRAIL expression in human and mouse pDCs [92,94,95,96]. Furthermore, pDCs can kill target cells by releasing the serine protease granzyme B (GrB), which is constitutively expressed in human pDCs [97]. The GrB production in human pDCs is positively regulated by IL-21 and is counteracted by autocrine production of I-IFN [98]. Although, the large majority of peripheral blood and tissue pDCs are CD56^-^, a small subset of circulating pDC-like cells has been found to express CD56, a marker specific for NK cells and associated with cytolytic effector function [14,99,100].

The major function of DCs is to capture antigens in the peripheral tissues and transport them to lymph nodes, where they are presented to T cells for efficient initiation of T cell-dependent immune responses [101]. pDCs display an antigen-presenting function, although lower compared to conventional mDC subsets. In addition to producing I-IFN, activated pDCs undergo morphological, phenotypic and functional changes. In vitro, pDCs acquire a dendritic morphology, up-regulate MHC-II and -I and co-stimulatory molecules (e.g., CD40, CD80, CD86) enabling antigen presentation to CD4^+^ T cells [7,102] and cross presentation to CD8^+^ T cells [103,104,105] even if less efficiently than cDCs. Furthermore, when pDCs mature into DCs their capability to produce I-IFN is lost [106,107]. Establishing which receptors contribute to antigen uptake and presentation by pDCs still remain an open question, although BDCA-2, DCIR, BST-2 and FCRII represent potential candidates [7]. Antigens coupled to antibodies that target these endocytic receptors are processed and presented to CD4^+^ T cells [63,72,108]. However, the crosslinking of BDCA-2 and DCIR triggers a signaling cascade that inhibits I-IFN production in pDCs suggesting that the primary role of these receptors is to trigger immunomodulatory signals, instead of capture antigens [63,64,72]. Unlike cDCs, pDCs are poor in the uptake of exogenous antigens, but they are able to sustain presentation of endogenous peptide [102]. In conclusion, pDCs are complementary to cDCs in their antigen presentation function.

Unstimulated or alternatively activated pDCs are able to induce T cell tolerance to tumor cells, harmless antigens and alloantigens, primarily through the induction of regulatory T cells (Tregs) [109,110,111,112]. pDCs induce tolerance by expressing indoleamine 2,3-dioxygenase (IDO) [113,114,115], inducible T cell costimulator ligand (ICOSL) [109], tumor necrosis factor ligand superfamily member 4 (TNFSF4; also known as OX40L) [116,117], PD-L1 [118] or GrB [119]. It has been reported that a fraction of pDCs found in tumor draining lymph nodes (TDLNs) express IDO and activate Tregs [113,115]. In addition, the expression of ICOSL on pDCs promotes the generation of IL-10-producing Tregs from naïve T cells [109]. IL-3 can induce OX40L up-regulation in pDCs. Hence, pDCs can modulate T cell responses via OX40L-OX40 interaction and Th2 polarization [117]. Finally, pDCs can suppress T cell proliferation by GrB secretion [119]. GrB secretion is enhanced by IL-3 and IL-10 and is inhibited by TLR and CD40L signaling.

## 3. Plasmacytoid Dendritic Cells and Cancer

### 3.1. Murine Pre-Clinical Models

A better understanding of the role of pDCs in cancer has been limited by the absence of appropriate murine models lacking this cell type. It is well-recognized that I-IFN is a critical component of the cancer immunoediting process in mice [120]. The endogenous IFN-α/β prevent the growth of carcinogen-induced and transplantable tumors and are required for the rejection of highly immunogenic methylcholanthrene-induced sarcomas [120]. In vivo studies, analyzing the host anti-tumor response, in the absence of pDCs, are limited to the transplantable B16 melanoma, a poorly relevant melanoma model from a translational point of view. However, in this system, topical administration of TLR-agonist induced tumor regression in established B16 melanoma through the recruitment of pDCs [95,121]. Furthermore, the intra-tumor injection of CpG-activated pDCs induces the recruitment and activation of NK cells and cross-priming of tumor antigens-specific cytotoxic T lymphocytes (CTLs) [121].

The first attempts to deplete pDCs by using antibodies (Gr-1, 120G8, mPDCA-1 and 927) have been reviewed by Swiecki and Colonna [3]. These antibodies, not only deplete pDCs, but also additional immune cell types [122,123]. Relevantly, CD317 (also known as BST2) antibodies have been recently tested for in vivo depletion of pDCs in an immunocompetent transgenic mouse model of head and neck squamous cell carcinoma (HNSCC) [124]. BST2 is expressed on murine pDCs and plasma cells in steady-state conditions. Furthermore, it is strongly induced on different cell types, including tumor cells by type I and II IFNs [122]. Notably, the injection of anti-CD317 resulted in severe pDCs depletion and a significant delayed tumor growth [124]. Nevertheless, it should be considered that depletion of other BST2-expressing cells could occur in this model system.

A transgenic mouse that expresses the diphtheria toxin receptor (DTR) under the control of pDC-specific gene promoter (i.e., the BDCA-2 promoter) [33] has been generated in the laboratory of Marco Colonna, following a similar approach to the CD11c-DTR transgenic mice [125]. The administration of diphtheria toxin (DT) to BDCA-2-DTR transgenic mice results in efficient and specific elimination of pDCs in blood and secondary lymphoid organs [126]. However, the resulting pDC depletion is transient and the pDC compartment is gradually restored over time, requiring repeated DT administrations. This unique transgenic mouse model has been successfully used to investigate the pDC functions in viral and bacterial infections [126,127,128], and in systemic lupus erythematosus (SLE) model [129].

In conclusion, the role of pDCs in cancer immunoediting is still poorly investigated, mainly due to the lack of appropriate model system for stable pDC manipulation.

### 3.2. Clinical Significance of the pDCs Compartment in Human Cancer Patients

**Figure 3:**
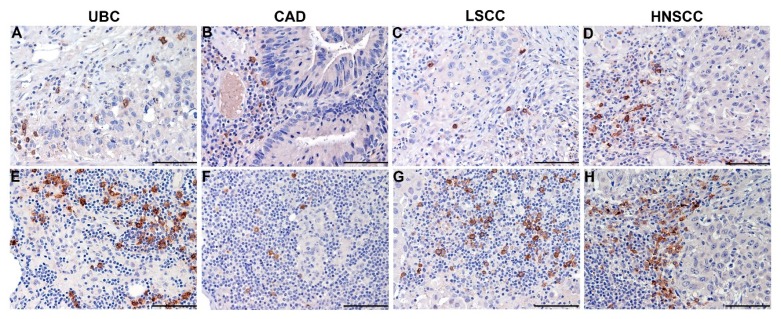
pDCs infiltration in primary carcinomas and carcinoma-draining lymph nodes. FFPE sections are from representative carcinoma cases from different primary sites (top row) and corresponding draining lymph nodes (bottom row). Matched images are from urothelial bladder cancer (UBC; **A**,**E**), colon-rectal adenocarcinomas (CAD; **B**,**F**), lung squamous cells carcinoma (LSCC; **C**,**G**) and head and neck squamous-cells carcinoma (HNSCC; **D**,**H**). Staining has been performed with BDCA-2 (clone 124B3.13, 1:75, Dendritics) and revealed using Novolink Polymer (Leica Microsystems) followed by 3,3′-diaminobenzidine (DAB) as chromogen. pDC infiltration is observed at the primary carcinoma sites (**A–D**) and is very dense in lymph node metastasis (**E–H**) suggesting that this rare cell subset can be found in a wide spectrum of solid tumors and corresponding draining lymph nodes. Magnification 200× (scale bar 100 µm).

Tumor associated-pDCs (TA-pDCs) have been detected in a wide variety of human neoplasms such as primary carcinomas from different primary sites (Figure 3), cutaneous melanoma and lymphomas. pDCs are located in the TDLNs of various cancer types, as well (Figure 3).

The recruitment of pDCs to tumor tissues is regulated by CKs secreted by neoplastic cells [15,46,130]. For example, the CXCR4/CXCL12 axis is involved in pDC migration toward melanoma, ovarian cancer and HNSCC [15,130,131]. In addition, the IFN-inducible expression of CXCR3 ligands (i.e., CXCL9, CXCL10, and CXCL11) sustains the migration of pDCs in response to CXCL12 [45,132,133]. Finally, we propose a role for Chemerin as chemotactic factor for tissue pDCs expressing the receptor ChemR23 [44], even if its relevance for pDC recruitment to tumor sites is still elusive.

The pDC recruitment has been documented in human primary breast cancer [134], and the corresponding metastatic sentinel lymph nodes (SLNs) in response to CXCL12/SDF1, including only a minor fraction with a mature phenotype [135,136]. pDCs were also observed in human ovarian carcinoma and malignant ascites from ovarian cancer patients [137,138,139]. Tumor-infiltrating pDCs have been identified also in HNSCC including the oral squamous cell carcinoma (OSCC) and in draining LNs, while they were almost absent in normal oropharyngeal mucosa [140,141,142]. On the contrary, lower numbers of tumor-infiltrating pDCs are found in colorectal cancer (CRC) compared to the adjacent normal mucosa, whereas the pDC/mDC ratio is increased in metastatic versus non-metastatic TDLNs [143].

Data on the frequency of circulating pDCs have been reported from cohorts of different cancer types. In general, although with some level of heterogeneity, circulating pDCs are decreased in human cancer patients compared to healthy donors, particularly in advanced stage diseases, with a single relevant exception in a study of non-small-cell lung cancer (NSCLC) [144]. Specifically, the peripheral blood pDCs resulted decreased in cohort studies from ovarian cancer [137], NSCLC [145], bladder cancer [146], CRC [147] and advanced breast cancer patients [148]. Notably, in ovarian cancer, circulating pDCs are partially restored after complete remission by chemotherapy [137]. Finally, the percentage of peripheral blood pDCs in HNSCC and OSCC patients was comparable to healthy donors [141,149].

The clinical significance of the density of the tumour-infiltrating pDCs and the frequency of circulating pDCs in human cancer has been analyzed in few studies. An increased pDC infiltration has been associated with poor outcome in breast cancer, ovarian cancer, NSCLC, OSCC and melanoma [138,141,150,151,152,153]. Of note, the density of TA-pDCs is increased in highly aggressive triple negative breast cancers showing worse prognosis [134,154], whereas another study showed a decrease of circulating pDCs in advanced breast cancer patients, suggesting that circulating pDCs could represent a positive prognosticator [148]. Furthermore, a high frequency of circulating pDCs correlates with prolonged overall survival in breast cancer and pancreatic cancer patients [148,155], while a higher MDSC/pDC ratio has been associated with poor survival in NSCLC [145]. Lack of correlation between the pDC density and the tumor stage is also reported in HNSCC [140].

Finally, the induction of Tregs by ICOSL^+^ pDCs has been reported in breast cancers, ovarian cancers, melanoma and liver tumors [138,143,154,156,157]. Of note, high densities of pDCs and ICOS^+^ Tregs are strong predictors of disease progression and early relapse in ovarian cancer [137,138,151].

### 3.3. The Dual Role of pDCs in Human Cancer

Properly activated pDCs are endowed with anti-tumor activity by inducing the apoptosis of neoplastic cells and secreting type I and type III IFN. However, the tumor microenvironment can subvert the anti-tumor function of pDCs. Tumor cells produce immunosuppressive cytokines (e.g., IL-10, TGF-β, PGE2), recruit Tregs, activate negative regulatory pathways (e.g., PD-1/PD-L1, CD80/CTLA4) or express immunomodulatory molecules (e.g., IDO), thus establishing an immunosuppressive microenvironment. Furthermore, TA-pDCs themselves can induce immune tolerance in many types of cancers, supporting the immunosuppressive milieu. For instance, TA-pDCs are mostly defective in their effector functions [10] and show an inhibitory phenotype. Several mechanisms could explain the pro-tumor role of pDCs in cancer, including the recruitment of I-IFN defective [15] and immature pDCs, as characterized by a lack of expression of co-stimulatory molecules [15,158,159], the promotion of tolerogenic functions by pDCs, such as the activation of mature Tregs, and the secretion of immunosuppressive factors by tumor cells.

Several studies have demonstrated that TA-pDCs display an immature phenotype, as shown by the lack of expression of the maturation marker CD83 and the co-stimulatory molecules CD80 and CD86 [15,158,159]. Moreover, pDC from cancer patients are defective in the production of IFN-α, IP-10, IL-6 and TNF-α. The reduction of IFN-α production by pDCs impairs the IFN-α-associated local immune response and promote immune-escape. The pDC dysfunction can be induced by soluble factors derived from tumor cells, necrotic cells or other immune cells, such as PGE2 [160], TGF-β [161], IL-10 [162], IL-3 [163,164], Vasoactive Intestinal Peptide [165], Wnt5a [166,167] and HMGB1 [168]. pDC dysfunction can also result from regulatory factors expressed by pDCs (e.g., IRF7, ILT7, IDO). The ILT7 ligand BST2, is endogenously expressed by a variety of human cancer cells [169] and inhibits I-IFN production by CpG [68,170,171]. However, limited data are available on BST2 expression in human cancer [10,172].

pDCs isolated from HNSCC tissues or exposed to HNSCC supernatants show an impaired IFN-α production upon CpG stimulation compared to blood-derived pDCs or pDCs exposed to control medium [131,140,173]. Tumor-induced down-regulation of TLR9 expression has been identified as one leading mechanism of pDC dysfunction within the tumor environment [140,142]. Furthermore, significant levels of PGE2, TGF-β and IL-10 are detected in the HNSCC and OSCC culture supernatants as mediators of IFN-α impairment in pDCs [140,142,161,173]. A decreased secretion of IFN-α, TNF-α, and IL-6 by tumor infiltrating pDCs was also detected in OSCC [141]. Similarly, in breast and ovarian cancers, TA-pDCs show a decreased IFN-α secretion upon TLR7/9 stimulation, but different soluble factors are involved, such as TNF-α and TGF-β. These cytokines act synergistically and impair IRF7-dependent IFN-α secretion [137,154,174].

Several studies have demonstrated that TA-pDCs might exert a tolerogenic function. For instance, pDCs induce the expansion and suppressive function of Tregs through the ICOS/ICOSL pathway or IDO expression [175] executing a pro-tumorigenic role [10,109,115]. The TA-pDCs and Tregs are co-localized in breast and ovarian carcinomas and their densities display a significant positive correlation [138]. The ICOS-driven interaction between tumor associated CD4^+^ T cells and ICOSL-expressing TA-pDCs sustain the expansion of ICOS^+^ FoxP3^+^ Tregs in the tumor microenvironment and promote the secretion of IL-10 and TGF-β from Tregs, which support an immunosuppressive microenvironment and tumor progression [137,138,150,151,152,154,174,176,177]. Furthermore, tumor-infiltrating pDCs, localized in close proximity to Tregs, are defective in IFN-α production. Finally, the pDC/mDC ratio correlates with FoxP3^+^ Tregs recruitment in metastatic TDLNs of CRC patients, further supporting that pDCs might contribute to Tregs development in the tumor milieu [143]. It should be reminded that, in addition to ICOS/ICOSL pathway, malignant cells and TA-pDCs release IDO, a potent activator of FoxP3^+^ Tregs [113,115,178,179,180,181].

Besides the induction of Tregs, pDCs might fulfill tumor-promoting functions through additional mechanisms. For instance, in ovarian cancer, pDCs modulate neo-angiogenesis via TNF-α and IL-8 production upon CD40L activation [139]. Recently, it has been demonstrated that TA-pDCs produce the pro-angiogenic and pro-invasive cytokine IL-1α in an AIM2-dependent manner, and promote tumor cell proliferation and angiogenesis in NSCLC [182]. Finally, the secretion of GrB could be an additional tolerogenic mechanism of pDCs [97,99]. GrB secretion is induced by IL-3, IL-10, and IL-21 and block CD4^+^ and CD8^+^ T cell proliferation [98,119]; on note, IL-21 has shown relevance in modulating cancer immunity [183].

In contrast to their pro-tumor functions, pDCs are endowed with an anti-tumorigenic capacity. Primarily, IFN-α production upon TLR-activation has direct and indirect effects on cancer cells and induce tumor regression, as shown in mouse model of orthotopic mammary tumor [121,184,185], becoming a critical component of the cancer immunoediting process [120,186]. Secondly, activated pDCs exert a tumoricidal activity against tumor cells via TRAIL and GrB [96,187]. CpG- or IMQ-stimulated pDCs are able to lyse in vitro TUBO breast cancer cell lines and to reduce the tumor burden in breast cancer [187]. Moreover, IMQ- or IFN-α-activated pDCs display a TRAIL-dependent cytotoxic activity against melanoma cell lines [96]. The role of TLR7 or IFNAR signaling in pDC-mediated cytotoxicity was confirmed by using mouse models of melanoma [92,95]. In addition, TRAIL^+^ pDC infiltration is observed in basal cell carcinoma lesions treated with the topical TLR7 agonist IMQ, suggesting that TLR-activated pDCs contribute to rejection of cutaneous tumors [92,188]. Finally, pDCs execute an antigen-presenting function in the tumor microenvironment, especially cross-presenting tumor derived antigens to CD8^+^ cytotoxic T cells [105]. Notably, they also enhance the mDC priming function, linking innate and adaptive immunity [185].

## 4. Human Cutaneous Melanoma: From Immunogenicity to Therapy

### 4.1. Melanoma as a Model of Hypermutated Hot Tumor

Cutaneous Melanoma (CM) is a neoplasm originated from skin melanocytes that progressively develop a malignant phenotype [189]. Several mutations occur during melanoma progression and the most commonly involved pathway is the mitogen-activated protein kinases (MAPK)/ERK cascade [190]. Mutations in the kinase domain of BRAF occur with a frequency around 50% in melanoma patients [191]. More than 80% of all BRAF mutations results in the V600E substitution that leads to constitutive kinase activation [191]. In about 15% of melanomas lacking BRAF mutation, the MAPK/ERK pathway is constitutively activated through mutation of NRAS, mostly at codon 61 [192].

Across human cancer types, CM is characterized by the highest prevalence of somatic mutations associated to UV signature [193,194,195,196]. The high mutational load makes melanoma a highly immunogenic solid tumor. Single nucleotide variation (SNV) and small insertions/deletions (indel) generate tumor-specific mutant antigens, playing a role in the recognition of cancer cells by the immune system. Cancer-specific mutant antigens are the target of checkpoint inhibitor-induced T cell responses [197] and their occurrence correlates with clinical response [198,199], and likely increase the recruitment of immune infiltrating cells. Particularly, indel mutations that cause a frameshift create a novel open reading frame (ORF) and could generate large amounts of tumor neo-antigenic peptides [200]. Accordingly, the indel load has been more strongly associated to checkpoint inhibitors response than SNV load in melanoma patients [196]. Notably, large-scale analyses of neo-antigen-specific T cell reactivity have been carried out for melanoma patients, showing that only a small fraction of non-synonymous mutations in expressed genes leads to the formation of neo-antigen for which CD4^+^ and CD8^+^ T cell reactivity can be detected within tumor-infiltrating lymphocytes.

Melanoma is considered an immunogenic “hot” tumor and its immune-mediated spontaneous regression has been documented [201]. A large fraction of Primary Cutaneous Melanomas (PCM) have high numbers of immune infiltrating cells, particularly proliferating T cells [202] mounting an antigen-specific response [203]. However, “hot” tumors might escape anti-tumor immune responses by up-regulation of checkpoint inhibitory ligands and secretion of immunosuppressive factors [204]. Whether specific mutated melanoma antigens are responsible for differences in the degree of tumor infiltration by lymphocytes is still under investigation [199,205]. Using The Cancer Genome Atlas (TCGA) dataset for CM [206], tumors have been categorized as T-cell-inflamed and non-T-cell-inflamed by gene expression profiling showing comparable numbers and in vitro immunogenicity of candidate neo-antigens [207]. By extending this study to other solid tumors, the lack of correlation between the tumor immune contexture and the load of mutational neo-epitopes was confirmed [207].

Contexture of the innate immune components is emerging as relevant in shaping the adaptive immune responses in PCM subtypes. Notably, BRAF^V600E^ PCM and distant metastasis exhibited an increased pDC numbers compared with BRAF-negative [208]. Moreover, we found a significant reduction in pDC density in NRAS^+^ and NF-1^+^ PCM, particularly when compared with the BRAF^+^ subgroup, whereas no differences were observed in CD8^+^ T-cell infiltration between TCGA subgroups [206,209]. Furthermore, Batf3-lineage DCs are critical for the induction of anti-tumor CD8^+^ T cell response. Analysis of the melanoma TCGA data indicates a strong correlation between CD8 gene transcripts and DC markers suggesting that lack of T-cell infiltration and activation is associated to failed recruitment and activation of Batf3 DCs [207].

By applying CYBERSORT method [210], tumor-infiltrating immune cell subsets, defined by their transcriptomic signatures, were associated with cancer outcome across 25 human malignancies [211]. Enrichment of T cell subsets correlates with superior survival, whereas dense myeloid populations predict poorer survival [211]. Moreover, CD8^+^ tumor-infiltrating lymphocytes expressing PDCD1 (encoding PD-1) and Cytotoxic T-Lymphocyte Antigen 4 (CTLA-4) correlate with response to checkpoint blockade therapy and survival in melanoma patients [212].

Remarkably, also melanomas responding to lymphocyte adoptive transfer or to checkpoint blockade display an increased mutational load and T cells specific for neoantigens [199].

### 4.2. Novel Systemic Treatments Partially Restrain Melanoma Dissemination

The therapeutic scenario of patients with inoperable or metastatic melanoma (MM) has been dramatically improved by the development of new treatment strategies. Before this modern era, historical dacarbazine was associated with a limited impact on patients’ prognosis and the median overall survival (OS) was about 6-9 months [213]. The introduction of inhibitors of MAPK pathway and blockers of immune checkpoint molecules has become the standard of care in advanced melanoma patients [214,215,216,217] providing a significant survival benefit.

Approximately one-half of melanoma harbors a BRAF mutation, mostly at codon 600: this molecular activation occurs early during melanoma evolution, driving both cancer growth and dissemination [191,218]. As firstly experienced in 2010 by Flaherty et al., mutated BRAF revealed its properties as a druggable target [219]. Specifically, highly selective BRAF inhibitors (BRAFi), such as vemurafenib or dabrafenib, improved outcomes for BRAF V600 MM patients, in comparison to cytotoxic chemotherapy [220,221]. Unfortunately, secondary resistance to BRAFi monotherapy represented the main source of treatment failure, limiting median progression-free survival duration to six months. The addition of MEK inhibitors (MEKi), enabling a double vertical inhibition of the MAPK pathway, reduced secondary resistance and decreased toxicity (such hyperproliferative skin lesion) typically associated to single-agent BRAFi [222,223,224]. Currently, the synergistic combination of a BRAFi plus a MEKi represents a standard of care in metastatic or locally advanced mutated melanoma. Several phase III randomized trials of first-line treatment, comparing BRAFi and MEKi (dabrafenib plus trametinib, vemurafenib plus cobimetinib, encorafenib and binimetinb) with single-agent BRAFi, showed that combinations were significantly associated to an advantage in both survival and response compared to BRAFi alone [222,223,224,225]. Moreover, the safety profiles were distinct and unique for either combination. The level of lactate dehydrogenase (LDH), the tumor burden and the number of metastatic sites are recognized as baseline prognostic biomarkers [226]. Notably, phase III trials exploring BRAFi plus MEKi combination regimens alone, in comparison to BRAFi, resulted in a greater survival benefit, regardless of prognostic factors [222,223,224,225]. Noteworthy, the coexistence of normal LDH level and a low tumor burden (less than three metastatic sites) were associated to a less aggressive disease and five-years OS and progression-free survival (PFS) rates from pooled analysis data using dabrafenib and trametinib [227]. Nevertheless, many patients did not achieve durable response even with the combination treatment, due to acquired resistance [222,223,224,225]. Recent advances are moving toward the identification of pre-existing tumor features predictive of target agents’ efficacy, besides BRAF mutation, to better personalize treatment approach [228].

Advances in the tumor immunology field has led to the development of inhibitors of immune-checkpoint molecules such as CTLA-4 and programmed cell death 1 (PD-1) [229]. Cancer immunotherapy relies on promoting anticancer immune response [230]. The complex interplay between cancer cells and host immunity contributes to the success of immunotherapy in metastatic and locally advanced melanomas. Pre-treatment cancer immune contexture (inflamed versus desert) predict response to anti-PD-1 or its ligand (PD-L1) [231]. Ipilimumab is a monoclonal antibody against CTLA-4 and the first clinical experiences were maturated in 2010. Specifically, the result of a pooled analysis of 1861 patients with MM treated with ipilimumab confirmed a plateau at 21% in the survival curve [232]. The subsequent development of selective immunosuppressive anti-PD-1/PD-L1 agents have further transformed the cancer immunotherapy scenario. Two phase-III studies (checkmate 067 and keynote 006), exploring the role of anti-PD-1 (nivolumab, and pembrolizumab, respectively), documented the improvement of survival and response in comparison to the control arm ipilimumab [233,234]. Recent update of both trials confirmed a five-years OS rate of 43% for pembrolizumab [235] and 44% for nivolumab [236], both as first line treatments; notably, these data revealed a plateau of the survival curves, as well. Moreover, immunotherapy was characterized by a frequency of tumor shrinkage from single-agent anti-PD-L1/PD-1 antibodies ranging from 10–40% [235,236].

Lately, the role of combination of checkpoint inhibitors has also been explored, revealing a synergic effect in term of survival and response. The phase III randomized trial checkmate 067 confirmed a statistically significant benefit of anti-PD-1 and anti-CTLA-4 (nivolumab plus ipilimumab) combination in comparison to ipilimumab and nivolumab alone [233,236]; specifically, the combination regimen resulted in a five-years OS rate of 52% with a median value not reached and a five-years PFS rate of 36% [236]. Survival advantage was confirmed regardless of BRAF mutation, LDH level or PD-L1 expression [236]. The overall response rate was 58% [236]. It should be noted that serious side effects and treatment discontinuation were more frequently represented in the combination arm than in monotherapy treatment with anti-PD-1 or anti-CTLA-4. Nevertheless, patients who discontinued combined treatment had a survival similar to patients belonging to the overall population [236].

Following these important advances in the treatment of MM, a new adjuvant therapy setting has emerged for high-risk stage III patients [237]. Target therapy and checkpoint inhibition immunotherapy have been explored in node-positive melanomas, at higher risk of recurrence after complete resection. The results of phase III randomized trials (keynote 054, checkmate 238 and combi-AD) demonstrated an improvement in recurrence-free survival in patients with resected melanoma, who received respectively anti-PD-1 (pembrolizumab or nivolumab) or target agents (dabrafenib plus trametinib, in BRAF mutated tumors) [238,239,240]. The rationale of these interventions was to eradicate the minimal residual disease and to reduce the risk of relapse.

Future challenges might involve the role of neo-adjuvant approach [241]. Both immunomodulating agents and target treatments have been explored in clinically stage III melanomas and preliminary results confirmed their potential therapeutic role [242,243,244].

### 4.3. The Immuno-Mediated Component of the Clinical Response to Systemic Treatments

The oncogenic BRAF can lead to immunomodulation and immune escape in melanoma, by i) the secretion of immunosuppressive cytokines in the microenvironment; ii) eliciting immune suppressive phenotype of the immune cells in the microenvironment; iii) modulating the MHC level in tumor cells [245].

The constitutive activation of MAPK pathway along with STAT3 pathway plays an essential role inducing the immune evasion by melanoma cells [246]. BRAF^V600E^ mutant melanoma cells produce immunosuppressive factors such as IL-10, IL-6 or VEGF [246,247]. These cytokines promote the recruitment of Tregs and myeloid-derived suppressor cells (MDSCs) in the tumor microenvironment and impair the production of IL-12 and TNF-α by LPS-stimulated DCs. DCs are critical for the induction of tumor-specific T-cell response and their impairment could explain the deficiency of T-cell response in melanoma. Thus, the MAPK pathway may represent a relevant molecular target for overcoming immune evasion in cancer. Furthermore, BRAF^V600E^ melanoma cells release IL-1 that up-regulates the transcription of several genes such as the co-inhibitory molecules PD-L1 and PD-L2 [248].

Another immunosuppressive effect of BRAF^V600E^ mutant proteins is related to the down-regulation of MHC-I molecules, via a rapid and constitutive internalization of MHC-I from the melanoma cell surface and subsequent sequestration within the endocytic compartments, thus diminishing CD8^+^ T-cell recognition of tumor cells and facilitating tumor evasion [249]. Inhibition of MAPK decreases MHC-I internalization, resulting in increased surface expression, which enhance melanoma antigen-specific CD8^+^ T-cell recognition and effector function [249].

Growing evidence suggest that for their therapeutic efficacy BRAFi and MEKi rely on factors that affect tumor-host interaction including the enhancement of melanoma antigen expression and the increase in immune response against tumor cells [250,251]. The mechanisms of intrinsic and acquired resistance to BRAFi and MEKi therapy have been previously reviewed [252]. Notably, immune-dependent mechanisms have also been recently identified. Among the latter, the expression of immunomodulatory molecules on the cell surface of tumor and T cells resulted increased within two weeks of therapy [250]. Moreover, tumor-associated macrophages contribute to the acquired resistance through the production of IL-1β [253].

Giving the high response rates of kinase inhibition and the response durability of immune checkpoint blockade, combinatorial approaches could be promising, boosting the immune responses and overcoming immune mediated mechanisms of resistance. These combinations have been tested in both preclinical models [254,255] and clinical trials (Clinicaltrials.gov study identifiers: NCT01400451, NCT01656642, NCT01767454, NCT02027961, NCT02130466). The scientific rationale for such strategies is based on the interplay between the MAPK pathway and the immune response in the tumor microenvironment. Pre-clinical and clinical observations indicate that inhibition of the MAPK pathway may have a favorable effect on the melanoma-specific immune response also at the level of T cells, DCs and tumor cells. The inhibition of BRAF and MEK leads to up-regulation of melanoma differentiation antigens (MDA), such as MART-1, gp-100 and tyrosinase, [256] and MHC expression [257] along with decreased immunosuppressive cytokines secretion [246]. Notably, an increased density of tumor-infiltrating lymphocytes (TILs) has been observed early after BRAFi treatment in metastatic tissue biopsies of MM patients [250,251,256,257]. Moreover, an increased expression of immune checkpoints (e.g., TIM3, PD-1 and PD-L1) in tumor samples from BRAFi-treated patients has been observed, suggesting a potential immune-mediated resistance mechanism [250] likely overcome by anti-PD-1/PD-L1.

Recent observations indicate that MEKi could negatively affect the human immune repertoire. In details, DC viability, cytokines secretion, expression of co-stimulatory molecules (e.g., CD40, CD80) and MHC-I are impaired by direct exposure of these cells to MEKi [247]. In addition, the T cell proliferative potential, their viability and IFN-γ production are reduced by in vitro treatment with MEKi [258]. However, in mice MEKi promote tumor-infiltration by CD8^+^ T-cells [259]. On post-treatment clinical samples, MEKi did not affect the frequency and phenotype of TILs [260]. All these findings should be properly considered when combining BRAFi/MEKi and checkpoint blockade in the treatment of melanoma.

In conclusion, MAPK inhibition might overcome the immune evasion of melanoma cells, enhancing DC function by several mechanisms.

## 5. Plasmacytoid Dendritic Cells and Melanoma

### 5.1. Clinical Relevance of Melanoma-Associated pDCs

Different studies have provided evidence that pDCs are part of the PCM immune contexture. While pDCs are almost absent in normal skin and melanocytic nevi, they are identified in PCM, predominantly located in the peritumoral areas admixed to lymphoid cells; more rarely, pDCs are found within the tumor nests in close association to melanoma cells [15,261]. A possible mechanism of pDC recruitment in human cutaneous melanomas is represented by CXCL12 [15] and CCL20, the latter identified as skin-homing CK [262]. pDCs have been abundantly identified also in the T-cell areas of SLNs, around HEVs, suggesting their derivation from the peripheral blood [15,263]. Higher frequencies of pDCs have been observed in positive SLNs compared to negative SLNs [263,264], and these pDCs are more immature, as shown by CD40 down-regulation [264].

**Figure 4:**
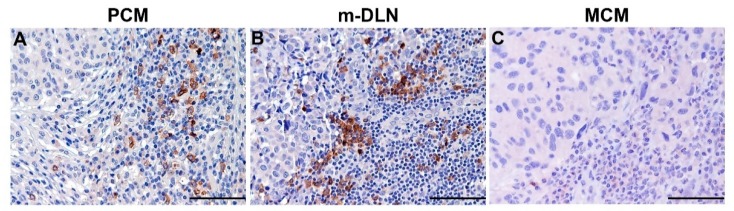
pDCs distribution in melanoma. FFPE sections stained with anti-BDCA-2 (clone 124B3.13, 1:75, Dendritics) and revealed using Novolink Polymer (Leica Microsystems) followed by 3,3′-diaminobenzidine (DAB) as chromogen. pDC infiltration is evident in primary cutaneous melanoma (PCM) (**A**) and melanoma-draining lymph nodes (m-DLN) (**B**), whereas the metastatic site (MCM) is devoid of pDCs (**C**). Magnification 200× (scale bar 100 µm).

**Figure 5:**
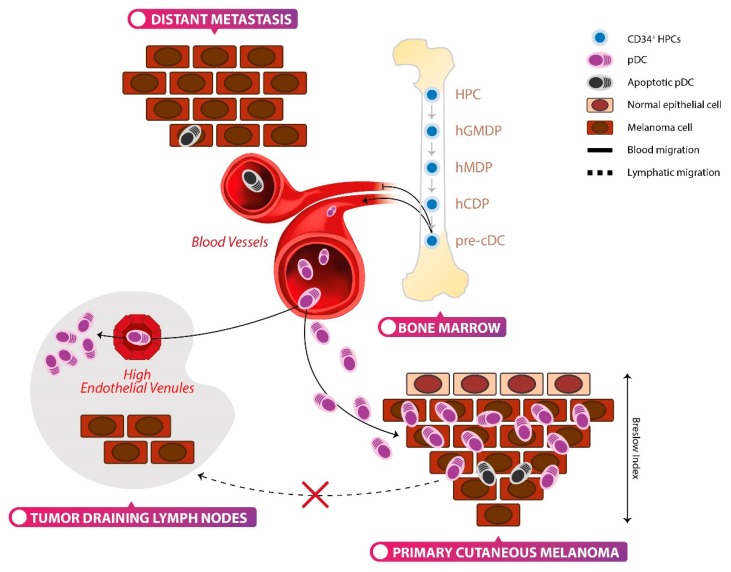
The pDC compartment in human cutaneous melanoma. Skin recruitment of pDCs from the peripheral blood is observed in Primary Cutaneous Melanoma (PCM) in the early phases of melanocyte transformation; over PCM progression, a reduced pDC density is coupled with increased Breslow thickness. Circulating pDCs efficiently home also to tumor draining lymph nodes, via high endothelial venules; on the contrary, PCM-infiltrating pDCs do not migrate via lymphatic vessels. pDC infiltration is dramatically decreased in distant metastasis of cutaneous melanoma. Based on in vitro experiments from our recent study [209], soluble factors impair pDC differentiation from the bone marrow progenitors and induce apoptosis of fully differentiated pDCs.

In line with the previous reports [15,261], we have recently documented that pDCs are recruited in the early stages of PCM, particularly at the invasive margin, whereas benign common-type nevi are mostly devoid of pDCs (Figure 4A). Moreover, we found that blood circulating pDCs efficiently home to draining LNs (Figure 4B) independently from their metastatic colonization, whereas PCM-infiltrating pDCs represent a non-migratory compartment [209]. However, a reduced pDC density occurs during local progression of PCM. More importantly, we could observe a dramatic decrease in pDC infiltration in distant metastasis (Figure 4C) combined with a collapse of circulating pDCs in chemo-naïve MM patients [209]. This finding was significant in advanced stage of disease (M1c group), characterized by visceral metastasis or high LDH serum level [209]. The pDC compartment is maintained by progressively restricted bone marrow progenitors [30] and an impairment in the pDC differentiation process might explain the pDC collapse in systemic disease. Our in vitro findings suggest that the exposure of CD34^+^ hematopoietic progenitor cells (HPC) and of terminally differentiated pDCs to soluble melanoma components reduces the generation of terminally differentiated pDCs and induces pDC apoptosis [209]. These findings are illustrated in the diagram of Figure 5.

It has been recently reported that the occurrence of BRAF mutation is associated to an increased pDC infiltration compared with BRAF-wild type melanomas [208]. Our findings further expand the notion showing that pDC density is significantly reduced in NRAS p.Q61-mutated PCM, likely depending on the limited ability of NRAS-mutated melanoma cells to chemoattract pDCs [209].

A small set of retrospective analyses has provided data on the prognostic significance of circulating and melanoma-infiltrating pDCs [157,265,266]. These findings are still inconclusive and conflicting. Systemic abnormalities of the pDC compartment have been described in melanoma patients, but their significant correlation with the variable of outcome is often not reported [157,209,267] (Table 1). pDC infiltration predicts poor prognosis in PCM and is strongly associated with phospho-STAT3 expression by melanoma cells, suggesting a tumor-induced immunosuppression mechanism [265]. Furthermore, melanoma-associated pDCs express high levels of IDO contributing to immune evasion [180]. On the other hand, the histological regression of melanocytic lesions has been associated with dense pDC infiltration and MxA expression (particularly in thin regressive melanomas), as indicator of endogenous I-IFN production. The IP-10/CXCL10 expression in PCM was also documented, promoting the recruitment of CXCR3^+^ and GrB^+^ lymphocytes [268].

By analyzing the levels of circulating pDCs in melanoma patients at different disease stages, a lower frequency of pDCs has been measured in patients with systemic spread (stage IV AJCC) compared to loco-regional disease. This decrease has been detected also in patients with active disease at the time of inclusion [266]. A decline in the pDC frequency was not only associated with a decreased PFS, but also occurred even before the relapse or progression was clinically detectable, suggesting its predictive value in the patient follow-up [266]. The frequency of circulating MDSCs was higher in patients with systemic disease and a significant inverse correlation was found between pDCs and MDSCs. pDC decline was the major negative prognosticator on OS and PFS in melanoma patients, independently of disease stage or frequency of other circulating cells [266]. A significant decrease in the absolute number of circulating pDCs in patients with cutaneous melanoma compared to healthy subjects was confirmed by another study, particularly in advanced stage disease [267]. On the contrary, in stage I melanoma an increased numbers of circulating pDCs is observed [269].

Aspord et al. found that within the CD45^+^ fraction a higher proportion of pDCs infiltrates cutaneous tumors compared with LN metastasis. In PCM, pDCs were positively correlated with the Breslow index and significantly associated with a poor clinical outcome [157]. On the other hand, the circulating component of pDCs decreased in the peripheral blood of advanced stage III-IV compared with early stage I-II melanoma. In the same study, patients with advanced melanoma showed higher percentages of circulating OX40L^+^ pDCs and Th2 T cells compared with patients at an early stage of the disease [157].

The differences observed in the clinical significance of the pDCs compartment found between studies are reported in Table 1. They likely depend on the tissues analyzed (primary versus metastatic), on the analysis method of the pDC frequency (digital microscopy of melanoma tissue versus flow cytometry of single cell suspensions) and how it is expressed (absolute versus relative values), as well as on the pDC markers (CD303 versus CD123) [270] used.

### 5.2. pDC Function in Human Cutaneous Melanoma

As aforementioned, proper pDC activation induces anti-tumor immunity, whereas pDCs conditioning by the tumor microenvironment mediate immune suppression. By using the human pDC cell line GEN2.2, it has been demonstrated that TLR7 and TLR9 stimulation can induce a TRAIL-mediated cytotoxic activity in pDCs that could participate to the tumor cell clearance [92]. In a murine melanoma model, the topical application of IMQ leads to TRAIL and GrB secretion and resulted in tumor clearance in a TLR7/MyD88- and IFNAR1-dependent manner [95]. However, the role of melanoma-infiltrating pDCs in tumor progression remains poorly explored.

The function of pDCs obtained from melanoma patients has been rarely investigated and the large majority of functional studies on PCM-infiltrating pDCs adopted surrogates of the main pDC functions. The expression of the IFN-α inducible protein MxA in PCM is very limited in the majority of the cases [15] and the poor IFN-α production by pDCs has been associated with melanoma growth [15,263]. Melanoma cells produce immunosuppressive cytokines, such as PGE2, IL-10 and TGF-β, that can inhibit TLR7/9 and IRF7 expression leading to a limited I-IFN production by pDCs [167]. Furthermore, nodal pDCs are functionally impaired in their IFN-α production [263] and favor immune tolerance via IDO expression [180]. Aspord et al. demonstrated that the activation status (i.e., co-stimulatory molecules expression) of pDCs is higher at the primary tumor sites compared to lymph node metastasis. Notably, pDCs isolated from both sites and from the peripheral blood of patients remain fully sensitive to TLR7/9 ligands stimulation in term of IFN-α and IP-10 production compared to healthy donors [157]. In addition, pDCs in the melanoma environment drive a pro-inflammatory Th2 response and increase the frequencies of IL-5, IL-13 and IL-10 producing T cells. The Th2 and the T-regulatory polarization is correlated with a high proportion of OX40L- and ICOSL-expressing pDCs [157]. Moreover, a subset of circulating pDCs expressing LAG-3 result alternatively activated via interaction with MHC-II to produce limited IFN-α and enhanced IL-6. These LAG-3^+^ pDCs are enriched at the melanoma sites and polarize toward an immune-suppressive environment [271]. In summary, the melanoma microenvironment might impair the I-IFN production by pDCs and induce their switch towards a tolerogenic function, thus promoting tumor growth. It should be reminded that the main function of pDCs (i.e., IFN-α production) in the circulating compartment of melanoma patients has been poorly investigated (Table 1) suggesting that a monitoring in clinically oriented cohort is mandatory. Understanding the balance between anti-tumor and tolerogenic functions of pDCs could pave the way for new therapeutic options in melanoma treatment.

### 5.3. Therapeutic Implications of Human pDCs in Melanoma

Although pDCs represent a minor population among circulating immune cells and in the tumor microenvironment, they represent a promising target for cancer immunotherapy (Table 2), based on their multiple networks with other immune cells.

The administration of recombinant IFN-α was an approved cancer immunotherapeutic approach, as monotherapy or combinational therapy [272]. However, the overall response rate resulted in quite low and serious grade toxicity reported [273]. Targeted delivery of IFN-α into the tumor environment enhance the local immune response and the benefit of the checkpoint inhibition, and reduce side effects [272]. Clinical trials combine intra-tumor injection of IFN-α with anti-PD-1 immunotherapy (Clinicaltrials.gov study identifier: NCT02339324), to overcome PD-L1-mediated escape [274]. Of note adjuvant IFN-α2b is associated with increased IDO expression by circulating pDCs and tryptophan consumption in the patients serum, suggesting that also this escape mechanism might dampen the clinical response [275].

It has been proposed that pDC functions are hijacked by the tumor microenvironment, but upon appropriate re-activation, they can be reprogrammed to anti-tumor functions. One of the possible approaches is the therapeutic activation of tumor-associated pDCs using TLR7 and TLR9 agonists. These compounds have shown some level of clinical benefit in antitumor immunity, through the production of I-IFN and cytotoxic molecules by pDCs [95,276,277,278]. Numerous clinical trials (phase I-III) administering different compounds are ongoing (Clinicaltrials.gov study identifiers: NCT02644967, NCT03445533, NCT03052205, NCT03084640, NCT03618641, NCT02680184, NCT03831295 and NCT02521870), even for patients with MM refractory to PD-1 blockade [279]. Antitumor effects of TLR9-targeted therapies have been described in animal models against various tumor types, including melanoma [280]. A phase II pilot trial has been carried out in twenty patients with unresectable IIIb/c–IV stage MM by subcutaneous injection of TLR9-stimulating oligodeoxynucleotide. In this study, increased activation of pDCs was documented by CD86 and MHC-II up-regulation, but changes in pDC counts were not evident [281]. Clinical activity of synthetic ODN injected in cutaneous or subcutaneous melanoma metastasis was also demonstrated in a phase I study on a small group of five patients [282].

Imiquimod (IMQ) is a synthetic TLR7 agonist approved by the FDA for the treatment of non-melanoma skin cancers (e.g., basal cell carcinoma). Aspord et al. investigated the IMQ effects using an innovative melanoma-bearing humanized mouse model. IMQ strongly inhibits melanoma tumor growth by i) prompt mobilization of pDCs; ii) triggering the pDCs cytotoxic functions and iii) up-regulation of expression of I-IFN-inducible genes [283]. In humans, clinical trials administering IMQ in combination with other therapies or tumor vaccines are ongoing. In detail, a phase II clinical trial combining topical IMQ with monobenzone in twenty-one melanoma patients (stage III–IV) led to the local regression of cutaneous metastases in 52% of the patients [284]. In addition, adjuvant FLT3L administration combined with a peptide-based vaccine and with topically applied IMQ was tested in eight patients with MM [285]. TLR7 agonist was used as a vaccine adjuvant in nine malignant melanoma patients, resulting well-tolerated and inducing tumor pDC infiltration [286].

Antigen-pulsed activated pDCs generate an antigen-specific CTL response against melanoma [185], encouraging the development of pDC-based anticancer vaccines. The use of pDCs as vaccination against cancer has been successfully accomplished for the first time in the laboratory of Jolanda De Vries. In a cohort of fifteen MM patients, autologous pDCs were activated and loaded with melanoma-associated peptides (gp100 and tyrosinase), and subsequently injected within the lymph node [287]. The pDC vaccine induces a systemic I-IFN response and activate NK cells. In addition, pDCs efficiently migrate to draining lymph nodes and activate nodal effector T cells. Of note, pDC vaccination resulted in increased OS compared to standard chemotherapy alone. Two additional preclinical studies support the development of a pDC-based vaccine for adoptive cellular immunotherapy in melanoma patients [288,289], showing that allogeneic pDCs loaded with melanoma antigens are potent inducers of tumor-specific T cell immunity [288,289].

In conclusion, properly activated pDCs could represent an addition on the existing and prospective treatments for melanomas.

## Figures and Tables

**Table 1:** Studies on the tumor-associated pDCs in melanoma patients.

Patients’ Sample (N°)	pDC Source	Detection Method	pDC Frequency	pDC Functional State *	Clinical Significance **	Reference
PCM (5)	FF	IHC; marker: CD123^+^ BDCA2^+^	Increased in PCM vs. sk/nevi	NA	NA	[261]
PCM (15); SLN^−^ (2); SLN^+^ (2)	FF; FFPE	IHC; marker: CD123^+^ BDCA2^+^	Increased in PCM vs. sk/nevi	Defective	NA	[15]
r-PCM (14)	CF	IHC; marker: BDCA2^+^	Increased in PCM vs. sk	Increased in early-intermediate vs. late r-PCM	Regression of melanoma	[268]
SLN^−^ (5); SLN^+^ (5); mLN (5)	FFPE	IHC; marker: CD123^+^	Increased in mLN	-	NA	[263]
SLN^−^ (19); SLN^+^ (5); mLN (5)	FF	IF; marker: BDCA2^+^	-	Defective	NA	[263]
SLN^−^ (31); SLN^+^ (8); mLN (9)	Cell suspension	FC; marker: CD123^+^ BDCA2^+^	Increased in SLN^+^/mLN vs. SLN^−^	-	NA	[263]
SLN^−^ (31); SLN^+^ (8); mLN (9)	Cell suspension	ELISA	-	Defective	NA	[263]
PCM (186)	FFPE	IHC; marker: CD123^+^	-	NA	Negative (PFS, OS)	[265]
SLN (36)	Cell suspension	FC; marker: CD123^+^ BDCA2^+^	Unchanged in stage I/III	NA	NA	[264]
PCM (397); SLN (71); MCM (25)	FFPE	IHC; marker: CD123^+^	Decreased in MCM vs. PCM; Increased in BRAF^V600E^ vs. BRAF^wt^	NA	None	[208]
PCM (12); mLN (28)	Cell suspension	FC; marker: CD123^+^ BDCA2^+^	Decreased in mLN vs. PCM	Functional	Negative (OS)	[157]
Stage I-IV melanoma	Blood	FC; marker: CD123^+^ BDCA2^+^	Decreased in stage III-IV vs. I-II	Unchanged	NA	[157]
PCM (101); SLN (33); MCM (60)	FFPE	IHC; marker: BDCA2^+^	Increased in PCM vs. nevi; Increased in SLN vs. PCM; Unchanged in SLN^+^ vs. SLN^−^; Decreased in MCM vs. PCM	NA	None	[209]
MM (29)	Blood	FC; marker: CD123^+^ BDCA2^+^	Decreased in MM vs. HD	NA	NA	[209]
Stage I and IV melanoma	Blood	FC; marker: CD123^+^	Increased in stage I vs. HD	NA	NA	[269]
Stage I-IV melanoma (69)	Blood	FC; marker: CD123^+^	Decreased in stage IV vs. stage I-III	NA	Positive (PFS, OS)	[266]
Stage I-IV melanoma (35)	Blood	FC; marker: CD123^+^	Decreased in PT vs. HD; Decreased in stage IV vs. I-II	NA	NA	[267]

* IFN-alpha production/MxA expression; ** prognosis associated to pDC frequency; NA = Not Assessed; PCM = Primary Cutaneous Melanoma; r-PCM = regressing Primary Cutaneous Melanoma; sk = normal skin; SLN^−^ = negative Sentinel Lymph Nodes; SLN+ = positive Sentinel Lymph Nodes; mLN = metastatic Lymph Nodes; MCM = Metastasis of Cutaneous Melanoma; MM = Metastatic Melanoma; PT = Patients; HD = Healthy Donors; FF = Frozen Fixed tissue; FFPE = Formalin Fixed Paraffin Embedded tissue; CF = Crio-fixed tissue; IHC = Immunohistochemistry; IF = Immunofluorescence; FC = flow cytometry.

**Table 2:** Therapeutic approaches using pDCs in melanoma patients.

Therapeutic Approach	Compounds	Type of Study	Number of Patients	Reference
IFN-α therapy + CPi	IFNα-2b + pembrolizumab	Phase 1	30 *	NCT02339324
IFN-α therapy + CPi	PegIFN-2b + pembrolizumab	Phase 1/2	293 *†	NCT02089685 [274,290]
TLR9-agonist + CPi	IMO-2125 + ipilimumab or pembrolizumab	Phase 1/2	53	NCT02644967
TLR9-agonist + CPi	IMO-2125 + ipilimumab	Phase 3	454 *	NCT03445533
TLR9-agonist	IMO-2125	Phase 1	54 †	NCT03052205
TLR9-agonist + CPi	CMP-001 + pembrolizumab	Phase 1	106 *	NCT03084640
TLR9-agonist + CPi	CMP-001 + nivolumab	Phase 2	32 *	NCT03618641
TLR9-agonist + CPi	CMP-001 + pembrolizumab	Phase 1	199 *	NCT02680184
TLR9-agonist + CPi	SD-101 + pembrolizumab	Phase 1/2	227 †	NCT02521870
TLR9-agonist	PF-3512676	Phase 2	20	[281]
TLR9-agonist	PF-3512676	Phase 1	10 †	[282]
TLR7-agonist	IMQ + monobenzone	Phase 2	25	[284]
TLR7-agonist + peptide-base vaccination	IMQ + NY-ESO-1	Phase 1	9	NCT00142454 [286]
pDC-based vaccination	HLA-A2.1^+^ pDC or mDC	Phase 1	30	NCT01690377 [287]
pDC-based vaccination	HLA-A0201^+^ pDC	Pre-clinical	12	[288]
pDC-based vaccination	HLA-A0201^+^ pDC	Pre-clinical	16	[289]

CPi = checkpoint inhibitor; * Estimated enrollment; † the study includes also non-melanoma tumors.
